# Sulbactam: A β–Lactam Compound with Neuroprotective Effects in Epilepsy

**DOI:** 10.3390/neurolint17090135

**Published:** 2025-08-27

**Authors:** Fang-Chia Chang, Chiung-Hui Liu, Wen-Chieh Liao, Yu-Shiuan Tzeng, Ru-Yin Tsai, Li-Ho Tseng, Ching-Sui Hung, Shey-Lin Wu, Ying-Jui Ho

**Affiliations:** 1 Department of Veterinary Medicine, School of Veterinary Medicine, National Taiwan University, Taipei 10617, Taiwan; fchang@ntu.edu.tw; 2 Neurobiology and Cognitive Science Center, National Taiwan University, Taipei 10617, Taiwan; 3 Graduate Institute of Acupuncture Science, China Medical University, Taichung 404328, Taiwan; 4 Doctoral Program in Tissue Engineering and Regenerative Medicine, College of Medicine, National Chung Hsing University, Taichung 402202, Taiwan; chiunghui.liu@gmail.com (C.-H.L.); khrnangel@nchu.edu.tw (W.-C.L.); 5 Department of Post–Baccalaureate Medicine, College of Medicine, National Chung Hsing University, Taichung 402202, Taiwan; 6 Department of Psychology, Chung Shan Medical University Hospital, Chung Shan Medical University, Taichung 402367, Taiwan; black125165@gmail.com; 7 Department of Anatomy, School of Medicine, Chung Shan Medical University, Taichung 402306, Taiwan; iris8084@csmu.edu.tw; 8 Graduate School of Environmental Management, Tajen University, Pingtung 90741, Taiwan; lhtzen@tajen.edu.tw; 9 Occupational Safety and Health Office, Taipei City Hospital, Taipei 10851, Taiwan; 10 Department of Neurology Changhua and Chang Bing Show Chawn Memorial Hospital, Changhua 500009, Taiwan; 11 Department of Neurology, Chang–Hua Christian Hospital, Changhua 500209, Taiwan

**Keywords:** epilepsy, astrocyte, glutamate, sulbactam, cognitive function, seizure

## Abstract

**Background:** The pathophysiology of epilepsy is characterized by increased neuronal activity due to an excess of the excitatory neurotransmitter glutamate and a deficiency in the inhibitory neurotransmitter gamma–aminobutyric acid (GABA). Epilepsy presents with seizures, neuronal loss, and hyperactivity in the subthalamic nucleus (STN). Astrocytes play a crucial role by absorbing extracellular glutamate through glutamate transporter–1 (GLT–1), thereby reducing neuronal excitation. Upregulating the expression of astrocytic GLT–1 is a promising therapeutic strategy for epilepsy. Sulbactam (SUL), a β–lactam antibiotic, has been demonstrated to exert neuroprotective effects by upregulating GLT–1 expression. **Objectives:** This study investigated the impact of SUL on neuronal and behavioral changes in epilepsy by using a pentylenetetrazol (PTZ)-induced rat model of epilepsy. **Methods:** Rats were treated with saline, SUL (50 and 150 mg/kg), or a combination of SUL and the GLT–1 blocker dihydrokainate (DHK) for 20 days. Subsequently, behavioral tasks were conducted to assess recognition, anxiety, and memory. **Results:** Histological analyses revealed that SUL ameliorated neuronal deficits, increased astrocytic GLT–1 expression, and reduced hyperactivity in the STN. Additionally, SUL promoted astrocyte proliferation, indicating a new dimension of its neuroprotective properties. However, the beneficial effects of SUL were prevented by DHK. **Conclusions:** This pioneering study highlights multiple benefits of SUL, including seizure suppression, increased GLT–1 expression, and astrocyte proliferation, underscoring its high potential as a treatment for epilepsy.

## 1. Introduction

Epilepsy is a heterogeneous neurological disorder characterized by recurrent, unprovoked seizures. It encompasses a wide spectrum of syndromes, which are broadly classified into focal and generalized types. Focal epilepsy originates from a specific region of the brain and may or may not spread to other areas, whereas generalized epilepsy involves widespread neuronal activity from the onset. Among focal epilepsies, temporal lobe epilepsy (TLE) is the most prevalent and clinically significant subtype, often associated with hippocampal sclerosis, cognitive deficits, and emotional disturbances [1,2,3]. Patients with TLE frequently exhibit reduced hippocampal volume and impaired memory and recognition, making it a relevant model for studying the neurobehavioral consequences of epilepsy and potential therapeutic interventions [4,5]. Deep brain stimulation targeting the subthalamic nucleus (STN) has been demonstrated to be partially effective in reducing seizures [6,7,8]; however, the role of STN in epilepsy is not yet clear.

An excess of excitatory neurotransmitters such as glutamate leads to increased neuronal activity, whereas a deficiency in inhibitory neurotransmitters such as gamma–aminobutyric acid (GABA) results in inadequate inhibition and increased hyperactivity [9,10]. Both conditions contribute to abnormal neuronal discharges and seizures [11,12]. Repeated seizures enhance excitatory synaptic transmission, potentiating NMDA-receptor-dependent Ca^2+^ influx and increasing neuronal excitability, indicating that seizures result in glutamatergic hyperactivity [13]. Therefore, reducing excitability while enhancing inhibition may help suppress seizures. One potential strategy involves removing excess glutamate and augmenting GABA production. Astrocytes play a critical role in this process because their glutamate transporter–1 (GLT–1) uptakes extracellular glutamate, thereby reducing neuronal excitability [14]. Furthermore, glutamate in astrocytes is converted by glutamine synthetase into glutamine [15], a precursor for GABA production. Studies have demonstrated that upregulating GLT–1 expression in astrocytes enhances glutamate removal, reduces excitotoxicity, and provides neuroprotection [16]. Therefore, we hypothesized that boosting GLT–1 expression in astrocytes protects neuronal and behavioral functions in epilepsy.

Sulbactam (SUL), a β–lactamase inhibitor, is commonly co-administered with antibiotics to prevent enzymatic degradation and enhance antimicrobial efficacy [17,18]. Research has demonstrated that SUL enhances GLT–1 expression in rat models of cerebral ischemia and substance abuse, having neuroprotective effects [19,20]. However, its effects on cognitive behavior, neuronal density, neurogenesis, and GLT–1 expression in an epilepsy rat model have not yet been thoroughly investigated. To validate the involvement of GLT–1 in SUL’s neuroprotective effects, dihydrokainate (DHK), a selective GLT–1 inhibitor, was included in this study.

The study employed a pentylenetetrazole (PTZ)-induced epileptic rat model. PTZ, a GABA receptor antagonist, inhibits chloride influx, increases neuronal activity, and lowers the seizure threshold [21,22]. The current study evaluated the behavioral and neurological effects of SUL in the PTZ-induced rat model of epilepsy.

## 2. Materials and Methods

### 2.1. Animals

In this study, 80 eight-week-old male Wistar rats (weight: 310 ± 35 g; BioLASCO Taiwan Co., Ltd., Yilan, Taiwan) were employed. They were randomly assigned to groups containing three or four individuals and housed in acrylic cages measuring 35 × 56 × 19 cm^3^. The housing conditions and handling procedures replicated those outlined in our previous study [23,24]. All experimental protocols adhered to the guidelines outlined in the NIH Guide for the Care and Use of Laboratory Animals and received approval from the Animal Care Committee of Chung Shan Medical University (IACUC Approval No. 2428, obtained on 30 December 2020). Stringent measures were implemented to minimize animal distress and reduce the overall number of animals used.

### 2.2. General Procedures

The rats were randomly assigned to two groups: the control group (*n* = 19) and the seizure group (*n* = 61). Kindling epilepsy was induced in the seizure group by administering intraperitoneal injections of PTZ (20–35 mg/kg) every other day from days 1 to 35. Specifically, the regimen involved an initial administration of two PTZ injections of 20 mg/kg and five PTZ injections of 30 mg/kg, followed by injections of 35 mg/kg PTZ. The rats in the control group received saline injections (1 mL/kg). The dose escalation protocol (2 × 20 mg/kg, 5 × 30 mg/kg, followed by 35 mg/kg) was adapted from other PTZ kindling studies [25]. This gradual increase ensures progressive kindling and reduces acute toxicity. The larger sample size in the epilepsy group was intended to accommodate subgroup stratification and potential attrition due to seizure-related mortality. As noted below, 11 rats died during seizures and 1 failed to meet the kindling criteria. The final subgroup sizes reflect these exclusions.

During the treatment phase (days 25–44), the rats received injections of either saline or drugs [SUL or dihydrokainate (DHK)]. The rats in the control group were given saline (1 mL/kg/day, IP; *n* = 19). In the seizure group, the rats were randomly subdivided into five subgroups: seizure + saline (*n* = 17), seizure + SUL50 (*n* = 9), seizure + SUL150 (*n* = 16), and seizure + SUL150 + DHK (*n* = 7). The treatments were saline (1 mL/kg/day, IP), SUL (50 or 150 mg/kg/day, IP), and DHK (0.14 mg/kg, IP), administered daily at 15:00. The SUL doses were selected on the basis of a study in which SUL was administered in combination with ampicillin (AMP) and showing that AMP + SUL (100 and 200 mg/kg) upregulated GLT–1 expression in the nucleus accumbens [26]. Additionally, AMP + SUL (200 mg/kg) reduced reinstatement to cocaine-seeking behavior and normalized GLT–1 expression in the mesocorticolimbic regions [27]. The appropriate SUL dose was estimated to be between 50 and 150 mg/kg.

**Figure 1:**
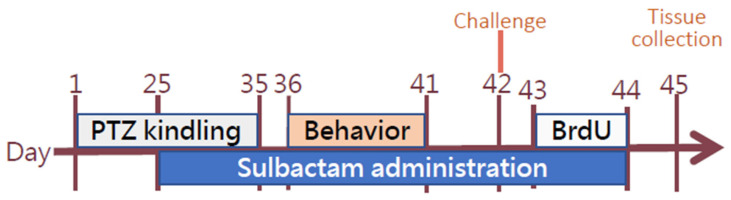
Experimental timeline of PTZ-induced epilepsy model and sulbactam intervention. The study spanned 45 days and included five major phases. From day 1 to day 35, rats received daily intraperitoneal injections of pentylenetetrazol (PTZ) to induce kindling. Sulbactam was administered from day 25 to day 44 to evaluate its potential neuroprotective effects. Behavioral assessments—including novel object recognition, elevated plus maze, and passive avoidance test—were conducted between day 36 and day 41. Bromodeoxyuridine (BrdU) was injected from day 43 to day 44 to label proliferating cells. On day 45, animals were sacrificed for tissue collection and subsequent histological assay.

Out of the 61 rats subjected to epilepsy induction, 1 rat failed to reach 4 points on the Racine score for two consecutive days, and 11 rats died during seizures, resulting in a mortality rate of 18%, significantly lower than the 40% reported in the literature [28]. Behavioral experiments were conducted from days 36 to 41 to assess object recognition (object recognition test), anxiety and movement (elevated–plus maze test; EPM), and memory (passive avoidance test; PAT). On day 42, a challenge test was performed with a PTZ injection (30 mg/kg). On days 43 and 44, the rats received intraperitoneal injections of 5–bromo–2′–deoxyuridine (BrdU; total dose 200 mg/kg, IP) for the labeling of newborn cells [29]. Following the experiments, the rats were euthanized on day 45 through CO_2_ exposure and then transcardially perfused with phosphate-buffered saline. Following this, their brains were fixed with 4% paraformaldehyde before being removed (Figure 1).

### 2.3. Kindling

PTZ, an inhibitor of GABA that hinders the binding of GABA to GABAA receptors, was employed to induce a rat model of kindling epilepsy [30]; procedures outlined in a previous study were followed [31]. A subconvulsive dose of PTZ (20–35 mg/kg, IP) was administered every other day from days 1 to 35. Following each PTZ injection, seizures and convulsions were observed in a sanitized cage (35 × 56 × 19 cm^3^) for 30 min. Seizure severity was assessed using the Racine score, reported in the literature [32,33].

### 2.4. Behavioral Tests

To evaluate rats’ recognition, anxiety, voluntary movement, and memory function, the following three behavioral tests were conducted in order. Prior to these experiments, the thorough cleaning of equipment and objects with 20% alcohol was performed, and all behavioral assessments were executed in a behavioral observation room featuring red light at 28 lux and white noise at 70 dB.

#### 2.4.1. Object Recognition Test

Rats inherently exhibit curiosity and spend more time exploring new objects in a familiar setting. The object recognition test harnesses this behavior to gauge their object recognition capability [34]. The recognition ability was assessed following procedures detailed in a previous report [23]. An open box measuring 60 × 60 × 60 cm^3^ was utilized, with objects A, B, C, and D fixed at three corners. Rats underwent three training sessions with a 24–hour interval. During training, each rat explored objects in the open box for 5 min. Five minutes after the last training session, a test session was conducted. The exploration time on the old object B during training and the new object D during the test was computed as a percentage of the total exploration time [(time spent on B or D/time spent on all objects) × 100%]. The discrepancy in the percentage of time spent exploring the old object B and the new object D served as a metric for recognizing familiar objects.

#### 2.4.2. Elevated Plus-Maze Test (EPM)

The anxiety-like behavior was assessed using the elevated plus-maze test [35]. The construction and testing procedures of the elevated plus-maze were consistent with our prior study [36]. Three measurements were recorded: (1) arm time, indicating the duration spent in open and enclosed arms, and (2) enclosed arm activity, measuring the frequency of the animal crossing a virtual line dividing an arm into proximal and distal halves.

#### 2.4.3. Passive Avoidance Test (PAT)

Memory retention was evaluated through the passive avoidance test, following our previous protocols [37,38]. The apparatus comprised light and dark compartments (20 × 20 × 20 cm^3^) with a metal grid floor connected to a shock scrambler. Rats were initially placed in the light compartment, and latency before entering the dark compartment was recorded. Upon entry into the dark compartment, a guillotine door closed, delivering an electric foot shock (0.5 mA, 3 s). The rat was then returned to its home cage. After 24 h, latency was measured again, termed retention latency. Importantly, no foot shock was administered when the rat entered the dark compartment.

#### 2.4.4. Histological Assessment

Sequential brain sections (25 μm thick; in the coronal plane) covering the hippocampus (from bregma −2.76 mm to −4.20 mm) and the STN were collected in accordance with the rat brain atlas [39]. Histological staining was conducted using methods reported in our previous paper [37,40]. Neuronal density in the hippocampus was evaluated using Nissl staining by following our established methodology [41,42]. Fluorescence immunohistochemistry was employed to assess GLT–1 expression in astrocytes marked by glial fibrillary acidic protein (GFAP) [41]. Neuronal activity in the STN was measured using the mitochondrial enzyme cytochrome c oxidase level [42]. Additionally, BrdU staining was used to detect newborn cells in the hippocampal dentate gyrus (DG) [23]. Primary antibodies included anti–BrdU (1: 1000; Cell Signaling, MA, USA), anti–GFAP (1: 300, Cell Signaling, MA, USA), and rabbit EAAT2 antibody (1: 300, GeneTex, CA, USA). Secondary antibodies were Alexa Fluor 448–conjugated Goat Anti–Mouse IgG (1: 500, Jackson, PA, USA) and Alexa Fluor 594–conjugated Goat Anti–Rabbit IgG (1: 500, Jackson, PA, USA).

Histological sections were captured using a microscope (ZEISS AXio Imager A2, ZEISS, Oberkochen, Germany). Images were analyzed using Image Pro Plus Software 6.0 (Media Cybernetics, Diego, CA, USA). The neuronal density in the hippocampal mCA3 and neuronal activity in the STN were quantified by following protocols established in our earlier studies. To determine the number of BrdU–labeled cells in the DG, we employed a semi-quantitative approach, based on stereological methods [43]. Exhaustive counting of the BrdU-labeled cells in the granule cell layer and subgranular zone of the DG was conducted. The absolute number of BrdU-labeled cells was then estimated by multiplying the counts by 6 because every sixth section was used. The figures obtained in our investigation represent absolute counts per DG.

### 2.5. Data Analysis

Statistical analysis was performed using SPSS (version 17.0). The Shapiro–Wilk test was used to assess normality and Levene’s test was used to evaluate homogeneity of variances prior to further test. Seizure severity data were analyzed using a mixed-design two-way analysis of variance (ANOVA). The results of the object recognition test were analyzed using a paired-sample *t* test, whereas those of the EPM test and PAT were analyzed using one-way ANOVA. Moreover, one-way ANOVA was employed to analyze the neuronal density in mCA3 and DG, the number of newborn cells in DG, neuronal activity in the STN, and the numbers of astrocytes and GLT–1-positive astrocytes. Post hoc testing was conducted using the least significant difference (LSD) test. Results are presented as the mean ± standard error of the mean (SEM), and the significance level was set at *p* < 0.05, unless otherwise indicated.

## 3. Results

### 3.1. Seizure Severity Score

A significant difference in seizure severity score during the kindling induction phase (days 1–25) between the control + saline and seizure + saline groups [F(1, 69) = 264.325, *p* < 0.001] was observed. During the treatment phase (days 25–35), a two-factor mixed-design ANOVA was conducted, which indicated no significant effects of treatment or time on seizure severity. Additionally, the day 42 challenge test revealed no significant differences in seizure severity between the groups (*p* = 0.346).

### 3.2. Object Recognition Test

**Figure 2:**
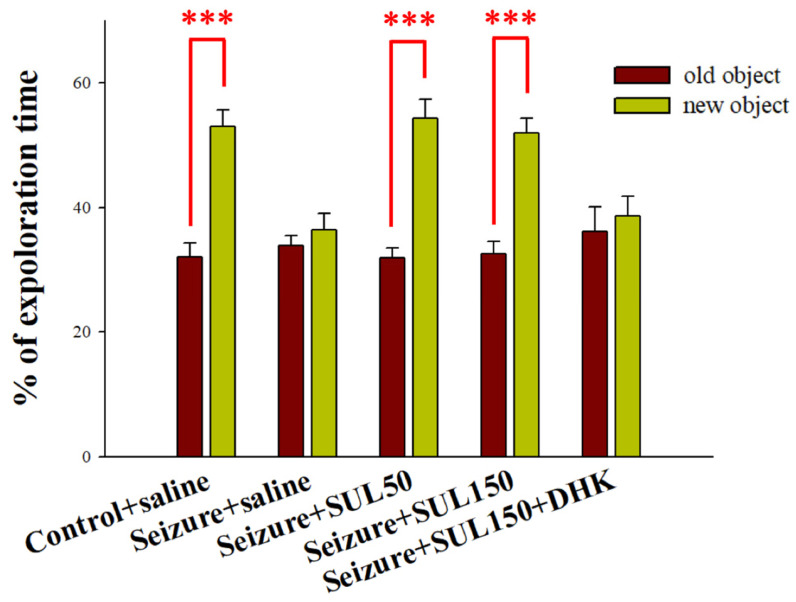
Effects of sulbactam (SUL) on behavior in the object recognition test in the pentylenetetrazol (PTZ)-induced kindling epilepsy rat model. *** *p* < 0.001, compared with the percentage of time spent exploring the old object. Data are presented as mean ± standard error of the mean (SEM).

The rats in the control + saline group spent significantly more time exploring new objects than they did exploring old objects (*df* = 18, *t* = −7.441, *p* < 0.001). By contrast, the rats in the seizure + saline group exhibited no significant difference in the time spent exploring new versus old objects (*df* = 15, *t* = −0.853, *p* = 0.407). Similarly, the seizure + SUL150 + DHK group did not exhibit a significant difference in their exploration time between new and old objects (*df* = 6, *t* = −0.472, *p* = 0.654). Notably, the rats in the seizure + SUL50 (*df* = 7, *t* = −8.378, *p* < 0.001) and seizure + SUL150 (*df* = 15, *t* = −5.898, *p* < 0.001) groups spent a significantly greater percentage of their time exploring new objects than they did exploring old ones (Figure 2).

### 3.3. PAT

**Figure 3:**
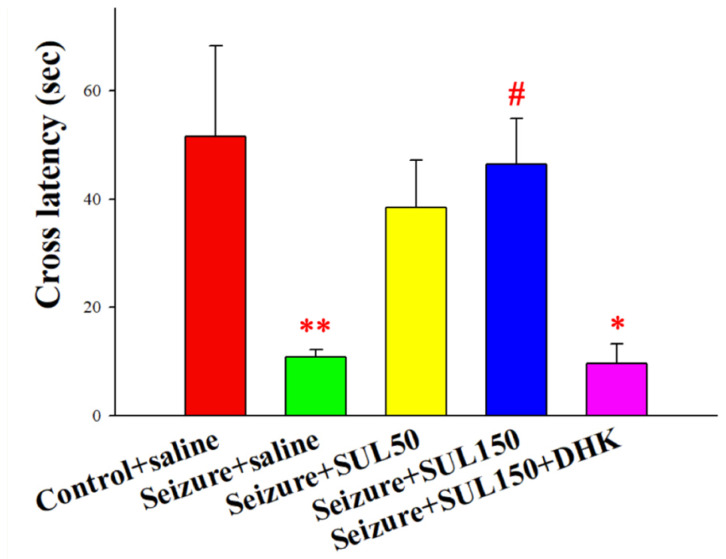
Effects of SUL on latency to enter the dark box in the passive avoidance test in the PTZ-induced kindling epilepsy rat model. * *p* < 0.05 and ** *p* < 0.01, compared with the control + saline group. # *p* < 0.05, compared with the seizure + saline group. Data are presented as mean ± SEM.

Significant intergroup differences in the latencies of rats moving from the light box to the dark box in the PAT [*F*(4, 53) = 3.219, *p* < 0.05] were observed. The results of the LSD post hoc test revealed lower entering latencies in the seizure + saline and seizure + SUL150 + DHK groups than in the control + saline group (both *p* < 0.05). Additionally, the entering latency of the rats in the seizure + SUL150 group was significantly higher than that of the rats in the seizure + saline group (*p* < 0.05) (Figure 3). Although the seizure + SUL50 group showed a trend toward improved latency, the difference did not reach statistical significance. The dose-dependent effects of SUL suggested that 150 mg/kg may be more effective in reversing memory deficits.

### 3.4. EPM Test

**Figure 4:**
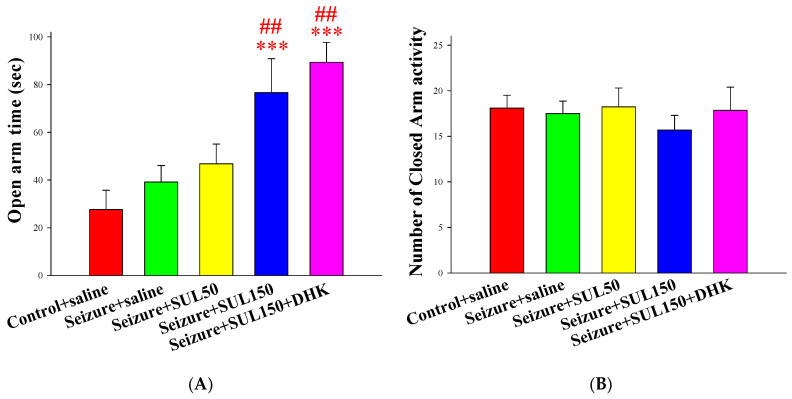
Effects of SUL on behavior in the elevated-plus maze test in the PTZ-induced kindling epilepsy rat model. (**A**) Open arm time. (**B**) Number of closed-arm activities. *** *p* < 0.001, compared with the control + saline group. ## *p* < 0.01, compared with the seizure + saline group. Data are presented as mean ± SEM.

The results showed significant intergroup differences in the time spent in the open arms of the EPM test [*F*(4, 56) = 5.904, *p* < 0.001]. The results of the LSD post hoc test indicated that the rats in the seizure + SUL150 and seizure + SUL150 + DHK groups spent significantly more time in the open arms than the rats in the control + saline (both *p* < 0.001) and seizure + saline (both *p* < 0.01) groups did. No significant intergroup difference was discovered in terms of closed-arm activity (*p* = 0.781) (Figure 4).

### 3.5. Neuronal Density in the Hippocampus

**Figure 5:**
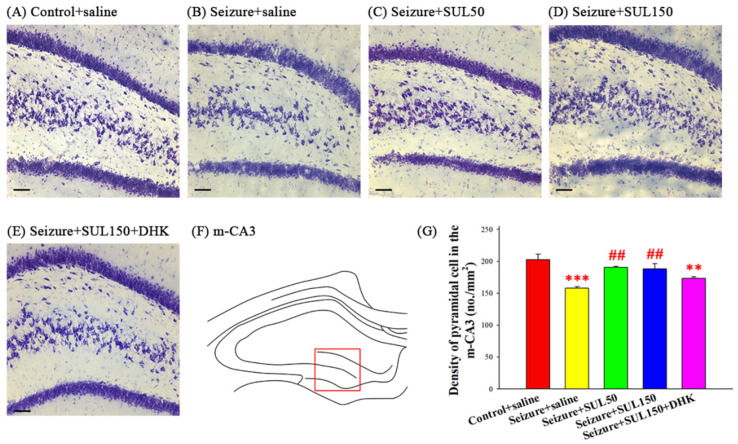
Effects of SUL on neuronal density in the hippocampus in the PTZ-induced kindling epilepsy rat model. (**A**–**E**) Coronal sections depicting Nissl stained cells in the medial CA3 (m–CA3) of the hippocampus for each group. Magnification, 100×; scale bar, 100 μm. The square in (**F**) denotes the region of m–CA3 used for measuring pyramidal neuron density. (**G**) Quantitative results (No./mm^2^). ** *p* < 0.01 and *** *p* < 0.001, compared with the control + saline group; ## *p* < 0.01, compared with the seizure + saline group. Data are presented as mean ± SEM.

One-way ANOVA with Brown–Forsythe correction revealed significant intergroup differences in neuronal density in the m–CA3 of the hippocampus between the groups [*F*(4, 8.336) = 13.741, *p* < 0.001]. The results of the LSD post hoc test revealed significantly lower neuronal density in the seizure + saline and seizure + SUL150 + DHK groups than in the control + saline group (both *p* < 0.01). Additionally, the neuronal density in the seizure + SUL50 and seizure + SUL150 groups was significantly higher than that in the seizure + saline group (both *p* < 0.01) (Figure 5).

### 3.6. Cytochrome C Oxidase in the STN

**Figure 6:**
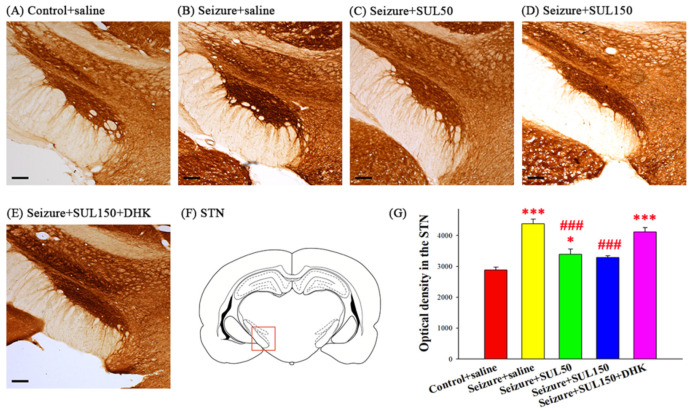
Effects of SUL on the concentration of cytochrome c oxidase (COX) in the subthalamic nucleus (STN) in the PTZ–induced kindling epilepsy rat model. (**A**–**E**) Depiction of COX staining in the STN through representative coronal sections for each group. Magnification, 50×; scale bar, 200 μm. The square in (**F**) marks the region of STN used for measuring COX concentration. (**G**) Quantitative results of optical density. * *p* < 0.05 and *** *p* < 0.001, compared with the control + saline group; ### *p* < 0.001, compared with the seizure + saline group. Data are presented as mean ± SEM.

The COX concentration in the STN was significantly different between the groups [*F*(4, 18) = 32.293, *p* < 0.001]. The LSD post hoc test indicated significantly higher COX concentrations in all seizure groups (seizure + saline, seizure + SUL50, and seizure + SUL150 + DHK) than in the control + saline group (all *p* < 0.05). However, the rats in the seizure + SUL50 and seizure + SUL150 groups exhibited significantly lower COX concentrations than the rats in the seizure + saline group (both *p* < 0.001) (Figure 6).

### 3.7. BrdU-Positive Cells

**Figure 7:**
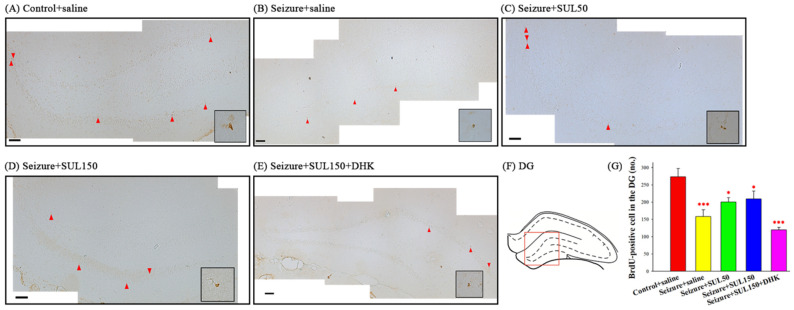
Effects of SUL on BrdU-positive cells in the hippocampal dentate gyrus (DG) in the PTZ-induced kindling epilepsy rat model. (**A**–**E**) Newly generated cells are indicated by BrdU labeling in representative coronal sections of each group. Magnification, 200×; scale bar, 100 μm. High-magnification insets (1000×) display BrdU-positive cells. The square in (**F**) indicates the DG area used for quantification. (**G**) Quantitative results (no). The red arrows in the figure point to new born cells. * *p* < 0.05, *** *p* < 0.001, compared with the control + saline group. Data are presented as mean ± SEM.

Significant intergroup differences in the number of BrdU-positive cells in the DG of the hippocampus between the groups [*F*(4, 12) = 9.037, *p* < 0.001] were observed. The LSD post hoc test revealed that the number of BrdU-positive cells was significantly lower in all seizure groups—seizure + saline, seizure + SUL50, seizure + SUL150, and seizure + SUL150 + DHK—than in the control + saline group (all *p* < 0.05) (Figure 7).

### 3.8. GLT–1 Expression in the Hippocampus

**Figure 8:**
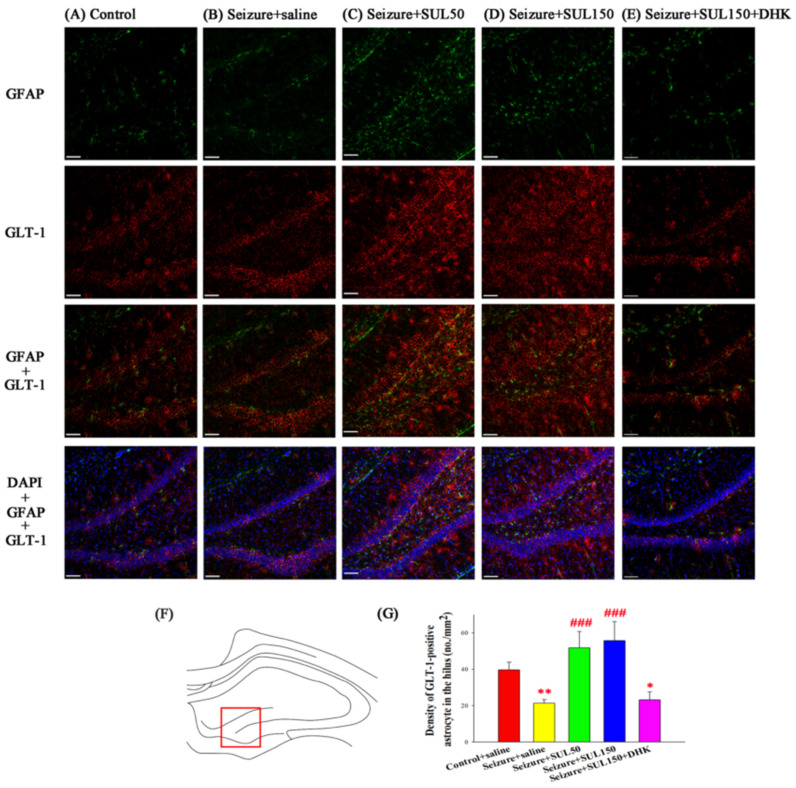
Effect of SUL on the density of astrocytes, GFAP-positive and GLT–1 expression in the hilus hippocampus in the PTZ-induced kindling epilepsy rat model. (**A**–**E**) Astrocytes are indicated by green GFAP labeling in representative coronal sections of each group. GLT–1 and pyramidal cells are shown in red and blue, respectively. Magnification, 200×; scale bar, 100 μm. The square in (**F**) indicates the hilus area used for quantification of astrocytes. (**G**) Quantitative results of astrocytes and GLT–1-positive astrocytes (No./mm^2^), respectively. * *p* < 0.05 and ** *p* < 0.01, compared with the control + saline group; ### *p* < 0.001, compared with the seizure + saline group. Data are presented as mean ± SEM.

The density of GLT–1-positive astrocytes in the hilus was significantly different between the groups [*F*(4, 16) = 8.846, *p* < 0.001]. The LSD post hoc test revealed a lower density of GLT–1-positive astrocytes in the seizure + saline and seizure + SUL150 + DHK groups than in the control + saline group (both *p* < 0.05). However, the seizure + SUL50 and seizure + SUL150 groups had a significantly higher density of GLT–1-positive astrocytes than the seizure + saline group (both *p* < 0.001) (Figure 8).

## 4. Discussion

In this study, kindling epilepsy was successfully induced in rats through intermittent PTZ administration. Compared with the control group, the epilepsy group (seizure + saline) and seizure + SUL150 + DHK group exhibited cognitive impairments in behavioral tests. Additionally, these groups exhibited hippocampal neuronal loss, increased activity in the STN, reduced neurogenesis in the DG, and diminished GLT–1 expression in astrocytes in the hilus. However, treatment with SUL at a dose of 150 mg/kg significantly ameliorated most of these deficits, including behavioral and histological impairments, although it did not improve neurogenesis. These findings suggest the potential efficacy of SUL in the treatment of epilepsy.

### 4.1. Kindling Epilepsy Rat Model

The literature has reported that rats with PTZ-induced epilepsy exhibit impaired motor function in the wheel test, abnormalities in the object recognition test, and a decline in memory in the PAT [44,45]. Moreover, reductions in the number of newborn cells and neuronal density in the hippocampus were discovered, as well as an increase in apoptotic cells in the amygdala [33,37,46,47]. These behavioral and histological changes are similar to those observed in patients with TLE. On the basis of a previous report [25], the PTZ dosage was gradually increased during the induction phase, by 20–35 mg/kg every other day. Initially, the PTZ did not trigger seizures; however, tonic–clonic seizures occurred in the later phase of the induction period [48,49,50]. The Racine score, a standard measure of seizure severity, defines full kindling as animals exhibiting tonic–clonic seizures with scores of 4 or 5 for a minimum of two consecutive days [51,52,53,54]. In our study, after nine PTZ injections over 17 days, the average seizure severity score exceeded 4, indicating the successful induction of kindling epilepsy.

To assess whether SUL suppressed seizures, we injected PTZ every other day during the SUL treatment period to induce a seizure and then recorded the seizure score. No significant difference in seizure scores was observed between the groups, which was not as expected and is also reasonable: PTZ is a potent seizure-inducing agent capable of triggering seizures even in healthy rats at a high dose, let alone our epileptic rats. Therefore, future research investigating the efficacy of antiepileptic drugs should focus on measuring the frequency, duration, and severity of spontaneous seizures or detecting neural activity through EEG in epileptic rats rather than relying solely on inducing seizures through PTZ injection. Our recent EEG data showed that after SUL treatment, epileptic rats exhibited less spikes and lower seizure frequency, compared with saline-treated epileptic rats, supporting this notion (data are preparing for publication).

### 4.2. Behavior

The rat model of PTZ-induced epilepsy exhibited behavioral deficits similar to those observed in patients with TLE [55], characterized by cognitive impairments related to the limbic system [56]. Research suggests that rats in the kindling epilepsy model exhibit impairments in memory, recognition, and spatial navigation, as assessed using the PAT, object recognition test, and water maze test, respectively [31,57,58]. Our epileptic rats exhibited similar deficits.

Some patients with TLE also experience affective problems, such as depression and anxiety, which may be associated with pathological changes in the amygdala. The literature reports that the volume of the amygdala in patients with TLE can be as much as 10–30% lower than that of healthy individuals [59]. In addition, gliosis and neuronal apoptosis in this region contribute to abnormalities in cognitive and behavioral responses to emotional stimuli [33,60,61,62,63,64]. The effects of SUL on the amygdala need to be evaluated in future studies.

Despite the findings from earlier studies, no consensus on anxiety levels across various epileptic rat models has been achieved. Rats with epilepsy induced by repetitive electrical stimulation in the amygdala exhibit a shorter time spent in the open arms during the EPM test, suggesting high anxiety. However, recurrent seizures can lead to emotional desensitization, showing low anxiety levels [65]. Neurological deficits induced by seizures in the hippocampus may affect the processing and storage of emotional stimuli, disrupting the cognitive interpretation of emotions. Further research is necessary to reveal the mechanisms underlying emotional responses in epilepsy rat models [65,66,67,68,69,70].

The EPM test is a longstanding method for assessing anxious behaviors, with the open arm representing an aversive stimulus. High-anxiety rats typically spend less time in the open arms [71]. Our study demonstrated no significant difference in open arm time between the control and epileptic groups. However, treatment with SUL, whether or not in combination with DHK, resulted in an increased open arm time, indicating a reduction in avoidance behavior, which was not due to a change in motor activity because no differences in closed-arm activity were noted between groups. While an increased open arm time is traditionally interpreted as reduced anxiety, we proposed that SUL’s modulation of STN activity—via GLT–1 upregulation—may contribute to reduced avoidance behavior. This hypothesis is supported by the observed reduction in cytochrome c oxidase activity in the STN and aligns with studies suggesting STN involvement in emotional regulation. These finding raise interest in evaluating the role of SUL and GLT–1 in stress-related behaviors.

### 4.3. Histology and Behavior

The hippocampus, vital for cognitive functions [72], exhibits atrophy in patients with TLE, as has been revealed through magnetic resonance imaging [73,74]. This atrophy, associated with hippocampal sclerosis, contributes to the decline in learning and memory [75,76,77]. In cases of chronic epilepsy and recurrent seizures, notable cell loss is observed in the hippocampus, particularly in CA1 [78,79]. In the present study, the neuronal densities in CA1, l–CA3, and m–CA3 were lower in the epileptic group compared with the control group. Moreover, behaviors associated with the hippocampus were impaired in the epileptic group, consistent with the findings in the literature [33,37]. Neurogenesis in the hippocampus is crucial for cognitive functions [80,81]. Rats in the epileptic group had fewer newborn cells in the hippocampus, potentially contributing to their cognitive deficits. Similarly, mice with fewer newborn cells in the hippocampus exhibited poorer spatial learning in the water maze test [82]. Furthermore, astrocytes in the hippocampus play a crucial role in epilepsy. Gliosis, observed in the hippocampus of patients with TLE, leads to morphological and electrophysiological abnormalities. The decreased expression of GLT–1 and glutamine synthetase in astrocytes may impede glutamate recycling in the synaptic cleft, leading to hyperexcitability and seizures. Similarly, epileptic rats exhibit a decreased number of GLT–1-positive astrocytes, suggesting that astrocytes could be a target for epilepsy treatment [83,84,85,86].

### 4.4. Metabolic Neuronal Activity in the STN

The STN, an integral component of the cortico-baso-thalamal loop, is involved in epilepsy [87,88,89]. Deep brain stimulation in the STN reduces seizures [6,7,8]. In epileptic rats, hyperactivity in the STN was observed. By releasing glutamate, the STN modulates neuronal activity, which could lead to overexcitation of the neuronal loop and contribute to pathophysiological changes in epilepsy. Reducing STN hyperactivity has been suggested as a promising treatment approach for epilepsy [90,91,92]. It has been reported that GLT–1 expression in astrocyte increases glutamate recycling [93], which may participate in the SUL-induced suppression of STN hyperactivity. However, no significant difference in seizure severity was observed after SUL treatment. The reason may be that PTZ injection triggered seizures and reached the ceiling, resulting in no discernible difference in seizure severity between the experimental groups. Therefore, assessing the frequency of spontaneous seizures and seizure thresholds in a future study would provide further insights into the effect of SUL on seizures. Although not included in this study, we have recently conducted EEG recordings showing a reduced spike frequency in SUL-treated epileptic rats. These data are being prepared for publication.

### 4.5. Mechanism of SUL

Elevated glutamate levels play a pivotal role in the pathophysiology of epilepsy through the activation of NMDA receptors and the induction of Ca^2+^ influx, neuronal excitation, and apoptosis [94]. Our findings indicate that SUL enhances GLT–1 expression in astrocytes, potentially increasing glutamate reuptake, reducing extracellular glutamate concentrations, and mitigating epilepsy-associated alterations.

The scientific literature indicates the ability of certain β–lactam compounds, including ceftriaxone, cefixime, and clavulanic acid, to increase GLT–1 expression in astrocytes, providing neuroprotective advantages [23,37,41]. SUL, a compound with a β–lactam structure, commonly used clinically, activates the p38 MAPK signaling pathway, leading to a 1.5 to 2-fold increase in GLT–1 expression. This activation reduced neuronal excitability and had neuroprotective effects in an ischemic animal model [19]. Although it has been reported that SUL exerts its function through GLT–1, our current study focused on GLT–1 expression and behavioral outcomes. Future experiments involving the detection of upstream signaling pathways, for example, p38 MAPK, are necessary to validate the signaling cascade and enhance our mechanistic understanding.

Astrocytes play a dual role, being involved in both the recycling of glutamate and production of GABA precursors. They take up extracellular glutamate via GLT–1, which is converted into glutamine and subsequently transformed into GABA through an interaction with glutamate decarboxylase 65/67 [15,95]. Therefore, the use of SUL is suggested not only to reduce glutamate excitability but also enhance GABA inhibition. The two mechanisms could synergistically contribute to neuronal protection in the epileptic rat model. Although not included in the paper, our recent study showed that SUL increased the density of astrocytes but not pyramidal neurons and GABA cells in the hippocampus of control rats. These data are being prepared for publication.

### 4.6. SUL Effects

The epileptic rodents under study exhibited both behavioral and histological impairments, indicative of the multifaceted nature of their condition. The administration of SUL at varying doses of 50 and 150 mg/kg was found to result in a dose-dependent enhancement of memory and object recognition abilities. Concurrently, the epileptic rat model demonstrated notable neuronal deficits, including a reduced hippocampal neuronal density, hyperactivity in the STN, decreased numbers of newborn cells in the DG, and the impaired expression of GLT–1 in astrocytes. Remarkably, treatment with SUL effectively ameliorated all these histological alterations, except for the number of newborn cells in the DG. The observed lack of impact on neurogenesis may be explained below. PTZ-induced seizures occurring shortly before BrdU administration may acutely suppress neural progenitor cell proliferation, thereby masking the potential neurogenic effects of SUL. This interpretation is supported by several studies. Even a single subconvulsive dose of PTZ can significantly reduce cellular proliferation in the dentate gyrus, suggesting that acute seizure activity may exert an inhibitory effect on neurogenesis [96]. Similarly, PTZ-induced seizures modulate granule cell precursor proliferation in the adult hippocampus, and this effect is sensitive to the timing and pharmacological modulation of glutamatergic signaling [97]. These findings imply that seizure-induced alterations in the neurogenic niche may interfere with the detection of pro–neurogenic interventions such as SUL, particularly when BrdU labeling is administered shortly after seizure onset.

Notably, SUL exhibited a dual effect by increasing not only GLT–1 expression but also the count of GFAP-positive cells. GFAP serves as a biomarker for various cell types in the hippocampus, including astrocytes, newborn cells, and type I neural stem cells. Therefore, some GFAP-positive cells in the hippocampal hilus are suggested to be newborn cells, comprising both astrocytes and undifferentiated cells [98,99]. Further research is warranted to elucidate the mechanisms through which SUL augments the number of GFAP-positive cells and the potential role of these cells in the repair of damaged brain tissue. The observed restoration of cognitive function and neurological deficits in epileptic rats following SUL treatment is attributable to the normalization of astrocyte numbers and the enhanced expression of GLT–1 on astrocytes in the hippocampus. This mechanism is presumed to enhance glutamate reuptake, thereby maintaining physiological extracellular glutamate levels and preventing excitotoxicity-induced neuronal loss, ultimately leading to cognitive function recovery. In the current study, astrocyte proliferation was assessed via GFAP and GLT–1 immunostaining, while BrdU labeling was used separately for neurogenesis. Co-labeling with GFAP + GLT–1 + BrdU in future work would provide more definitive evidence of functional astrocyte proliferation.

The GLT–1 blocker DHK [100] effectively countered both the behavioral and neuronal benefits of SUL, underscoring the crucial role of GLT–1 in mediating the effects of SUL. Surprisingly, in the seizure + SUL150 + DHK group, the expression of GLT–1 was significantly lower than that in the seizure + SUL150 group, suggesting a dual effect of DHK: functional blockade of GLT–1 and inhibition of its expression. The sample size for each group was determined based on previous studies using similar PTZ-induced epilepsy models and GLT–1 modulation [33,37]. Due to ethical considerations and the high mortality rate associated with PTZ-induced seizures, we aimed to minimize animal use while maintaining statistical validity. We acknowledge that the seizure + SUL150 + DHK group had a smaller sample size (*n* = 7), which may limit statistical power. To comprehensively assess the efficacy of SUL and better understand its mechanism of action, future studies should incorporate a positive control group. Furthermore, exploring the interaction between astrocytes and GABAergic neurons in mediating the effects of SUL warrants further investigation to elucidate the full therapeutic potential of SUL in epilepsy treatment.

In conclusion, our study demonstrated the ability of SUL administration to upregulate GLT–1 in astrocytes and thereby mitigate neuronal and cognitive impairments in an epilepsy rat model. These findings underscore the potential of SUL as a treatment option for epilepsy. Our findings pertain to PTZ-induced epilepsy, which models aspects of generalized seizures. While SUL shows promise in this context, further research is needed to determine its efficacy across other epilepsy subtypes, such as focal or genetic epilepsies.

## Data Availability

No new data were created or analyzed in this study. Data sharing is not applicable to this article.
